# Using the Uganda National Panel Survey to analyze the effect of staple food consumption on undernourishment in Ugandan children

**DOI:** 10.1186/s12889-017-4576-1

**Published:** 2017-07-19

**Authors:** Michelle M. Amaral, William E. Herrin, Grace Bulenzi Gulere

**Affiliations:** 1 Department of Economics, University of the Pacific, 3601 Pacific Avenue, Stockton, CA 95211 USA; 2 Department of Economics and School of International Studies, University of the Pacific, 3601 Pacific Avenue, Stockton, CA 95211 USA; 3 Directorate of Statistical Coordination, Uganda Bureau of Statistics, Plot 9, Colville Street, Kampala, Uganda

**Keywords:** Uganda, Child nutrition, Stunting, Wasting, Staple foods, Longitudinal data, Logistic regression, Odds ratio, Random effects

## Abstract

**Background:**

The United Nations’ Millennium Development Goals Report, 2015, documents that, since 1990, the number of stunted children in sub-Saharan Africa has increased by 33% even though it has fallen in all other world regions. Recognizing this, in 2011 the Government of Uganda implemented a 5-year Nutrition Action Plan. One important tenet of the Plan is to lessen malnutrition in young children by discouraging over-consumption of nutritionally deficient, but plentiful, staple foods, which it defines as a type of food insecurity.

**Methods:**

We use a sample of 6101 observations on 3427 children age five or less compiled from three annual waves of the Uganda National Panel Survey to measure undernourishment. We also use the World Health Organization’s Child Growth Standards to create a binary variable indicating stunting and another indicating wasting for each child in each year. We then use random effects to estimate binary logistic regressions that show that greater staple food concentrations affect the probability of stunting and wasting.

**Results:**

The estimated coefficients are used to compute adjusted odds ratios (OR) that estimate the effect of greater staple food concentration on the likelihood of stunting and the likelihood of wasting. Controlling for other relevant covariates, these odds ratios show that a greater proportion of staple foods in a child’s diet increases the likelihood of stunting (OR = 1.007, *p* = 0.005) as well as wasting (OR = 1.011, *p* = 0.034). Stunting is confirmed with subsamples of males only (OR = 1.006, *p* = 0.05) and females only (OR = 1.008, *p* = 0.027), suggesting that the finding is not gender specific. Another subsample of children aged 12 months or less, most of whom do not yet consume solid food, shows no statistically significant relationship, thus supporting the validity of the other findings.

**Conclusion:**

Diets containing larger proportions of staple foods are associated with greater likelihoods of both stunting and wasting in Ugandan children. Other causes of stunting and wasting identified in past research are also confirmed with the Uganda data. Finally, the analysis provides clues to other possible causes of undernourishment in young children.

## Background

Much attention has recently been focused on progress toward achieving the 2015 Millennium Development Goals (MDGs) established by the United Nations’ Millennium Declaration in September 2000. The first of these goals, to eradicate extreme poverty and hunger, includes a target to halve between 1990 and 2015 the proportion of people who suffer from hunger. A recent United Nations report includes a discussion of progress made since 1990 in lowering the proportion of underweight children aged five or younger [1]. It documents much progress, with the proportion of underweight children falling by 11 percentage points to 14% through mid-2015. It also reports that, 25 years on, Southern Asia still has the largest percentage of underweight children (28%), but that sub-Saharan Africa has now surpassed Southeast Asia as the region with the second highest proportion (20%). The same report also notes that stunting, or inadequate height-for-age, of children age 5 or less is more prevalent than being underweight. Finally, it reports that since 1990 the number of stunted children in sub-Saharan Africa has increased by 33% even though it has fallen in all other world regions.

Most of the work on the extent of child undernutrition done since the 2000 Millennium Declaration has focused on estimating trends in stunting among pre-school children and describing the prevalence of underweight children [2–4]. Other work analyzes the risks and health consequences of maternal and child undernutrition [5, 6]. As a result, quite a bit of work explores ways to mitigate child malnutrition that range from individual to national and international intervention strategies [7–9]. These analyses of interventions include cost considerations, schooling, social safety nets, as well as agricultural and early childhood development programs [10, 11]. The political environment within which nutrition-related policy decisions are made has also been studied [12].

Attention has also focused on the causes of undernutrition in young children. Earlier work focused more on socioeconomic factors shows that socioeconomic background explains child body size differences better than ethnicity, and that child malnutrition is a function of socioeconomic inequality [13, 14]. More recently, low household wealth has been shown to affect stunting in Kenya, eastern Uganda, and Cambodia [15–17].

Most of the studies showing that parental education affects child malnutrition find that less educated parents, especially mothers, are more likely to have malnourished children [18, 19]. Interestingly, another paper counterintuitively suggests that, in Ghana, more education increases the likelihood of stunting [20]. Another study in Uganda shows that stunting is more likely with less educated mothers but only in rural areas. It also shows that stunting is more prevalent among boys [21]. Other work focused on sub-Saharan Africa, including East Africa and Uganda in particular, showed a greater prevalence of child malnutrition in rural areas [22–25]. Perhaps most obviously, many studies show that dietary practices also affect malnutrition. Infant feeding practices have been shown to affect stunting and wasting in India [26]. In addition, children’s diet has been associated with childhood malnutrition in the East Africa region [27, 28].

The World Health Organization (WHO) compiles an indicator of dietary diversity to shed light on how diet relates to malnutrition among infants and young children. Corresponding country profiles show a wide range of dietary diversity across the 46 countries for which data were available [29]. Related to this, an influential study using some of these data provides evidence that dietary diversity affects child nutritional status in a number of sub-Saharan African, Asian, and Latin American countries [30].

Our research focuses on the lack of dietary diversity in Uganda, where the WHO country profile shows that only 23.6% of Ugandan children aged 6–23 months have a minimally diverse diet. This is almost a full standard deviation below the 39.5% average for the 46 countries profiled. Related work by the Food and Nutrition Technical Assistance II program (FANTA 2) for Uganda confirms the WHO profile [31]. It reports that most households across the country consume vegetables only three times per week, fruits less than twice weekly and meat and eggs less than once (p. 43). It also reports that households spend most of their food budget on staples (p. 42), and it documents the wide variety of staples grown in Uganda, most of which are cereals, tubers, and pulses. While all these staples are rich in Vitamin C, they have very low concentrations of Vitamin A, iron, and zinc. The lack of iron is consistent with the report stating that 73% of Ugandan children are anaemic (p. 17), and that low levels of zinc in children may have some bearing on the prevalence of stunting (p. 19).

FANTA 2 also defines food security as having both physical and economic access to sufficient, safe, and nutritious food. Under this definition, the report suggests that 73% of Ugandan households are “food secure,” 21% are “moderately food insecure,” and only 6% are classified as “food insecure” (p. 34). Perhaps because Ugandans spend so much on nutrition-deficient staples even though the country is mostly food secure, its government has promoted dietary diversity as a means to mitigate micronutrient deficiencies with the 5-year Nutrition Action Plan (UNAP) implemented in 2011 [32]. In fact, UNAP defines food insecurity in part as the frequent consumption of staples lacking sufficient Vitamin A, iron, and zinc.

Our research uses the proportion of household food spending on staples to explain stunting and wasting among children aged 5 years or less in Uganda. This complements other work that uses the WHO indicator of dietary diversity to explain stunting and wasting in Ethiopian children [33]. To our knowledge, the effect of staples on the nutritional status of children has been studied only once in the African context [34].

## Methods

### Data

Table 1 reproduces a complete list of all common staples consumed in Uganda from the FANTA 2 report. It shows quite a bit of overlap in the staples consumed in different regions of the country. Because staples are consumed in different proportions in different regions of Uganda, and because the data we use include households from the entire country, our definition of staple foods includes all those identified in Table 1 except beans. We exclude beans because they are a rich source of protein, and thus not nutritionally deficient. Table 1 informs the construction of our principal research variable, the staple budget share (SBS), which is defined simply as spending on staple foods expressed as a percentage of total household food spending. SBS is derived from a concept called the staple calorie share (SCS), defined as the percentage of total caloric intake comprised of staple foods. An economic analysis using the SCS studied the incidence of hunger among rural poor in China [35]. The study notes that most researchers will not be able to determine carefully the SCS with generally available survey data, but explains why using the SBS is an adequate proxy. Table 2 reports SBS summary statistics and the expected sign of its estimated coefficient. The data on household food spending used to construct the SBS and the other variables we use come from three annual waves of the Uganda National Panel Survey (UNPS), which is part of the World Bank’s Living Standards Measurement Study (LSMS) [36–38].

**Table 1:** Primary staples most frequently consumed by region

Region	Primary Staples Consumed (bold refers to most frequently consumed in the region)
Southwest	**Matooke**, cassava, patatoes, beans, maize, bread
Western	**Cassava**, beans, patatoes, matooke, maize
Central 1	**Cassava**, matooke, beans, potatoes, maize
Central 2	**Cassava, potatoes**, matooke, beans, maize, bread
East Central	**Potatoes**, cassava, maize, bread, beans
Eastern	**Maize, cassava**, potatoes, sorghum, milet, matooke, bread
Teso	Sorghum, millet, cassava, potatoes, beans
Lango	**Cassava, beans**, maize, potatoes
West Nile	Maize, cassava, potatoes, beans
Acholi	**Maize**, cassava, beans
Karamoja	Maize, sorghum, beans, beer residue
Refugee Camps	Maize, cassava, beans

This table is reproduced from FANTA-2: The Analysis of the Nutrition Situation in Uganda with the permission of the Food and Nutrition Technical Assistance II Project (FANTA-2). It can be accessed at http://www.fantaproject.org/sites/default/files/resources/Uganda_NSA_May2010.pdf)

**Table 2:** Variable definitions and summary statistics

Number of obs (n): 6101			
Number of unique children: 3427			
Variable	Definition	Mean (Std. Dev.)	Expected Sign
Outcome Variables			
Stunting	binary variable =1 if child’s z-score is more than two standard deviations (2sd) below WHO reference mean for height for age	0.222^a^ (0.416)	---
Wasting	binary variable =1 if child’s z-score is more than 2sd below WHO reference mean for weight for height	0.031^a^ (0.175)	---
Underweight	binary variable =1 if child’s z-score is more than 2sd below WHO reference mean for weight for age	0.098 (0.297)	---
Covariates			
SBS	staple food spending as a percentage of total household food expenditures	43.743 (19.579)	(+)
Food insecurity	binary variable =1 if hh reported it did not have enough food in one or more months that year, 0 otherwise	0.331 (0.471)	(+)
Spending	Total household spending (100,000 UGX 2011)	52.580 (73.515)	(−)
Household head	binary variable =1 if hh head is male, 0 otherwise	0.791 (0.407)	---
Average age	The average age of individuals residing in the household	22.329 (6.465)	---
Household occupants	Number of individuals residing in the household	5.446 (2.663)	(+)
Percent female	Percent of adults in the household that are female	53.533 (19.180)	(−)
Urban	binary variable =1 if dwelling is in an urban area, 0 otherwise	0.131 (0.337)	(−)
Father present	binary variable =1 if father is present in the household, 0 otherwise	0.722 (0.448)	(−)
Mother present	binary variable =1 if mother is present in the household, 0 otherwise	0.876 (0.330)	(−)
Household head educated	binary variable =1 if household head completed at least one year of schooling, 0 otherwise	0.411 (0.492)	(+)
Male	binary variable =1 if child is male, 0 otherwise	0.503 (0.500)	---

^a^Full sample means. Stunting subsample means: ≤ 12 months 0.118, > 12 months 0.242, males 0.248, females 0.196. Wasting subsample means: ≤ 12 months 0.060, > 12 months 0.026, males 0.037, females 0.026

### Study setting, design, and sampling strategy

**Fig. 1:**
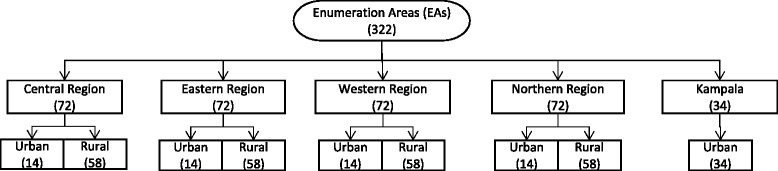
Sampling Procedure

The Uganda Bureau of Statistics (UBOS) followed and compiled a stratified random sample of households for three successive years between 2009/10 and 2011/12. These households are located in 322 enumeration areas (EAs) selected with equal probability across the country. UBOS enumerators then randomly chose ten households from each EA. The enumerators were able to follow 3123 households, or 9.7 per EA. Figure 1 shows the distribution of the 322 EAs. Thirty-four EAs are in Kampala, the capital and by far Uganda’s largest city. UBOS divides the remaining 288 evenly between each of Uganda’s four main geographic regions (Central, Eastern, Western, and Northern). Of these 72 EAs selected within each region, 58 are rural and 14 are urban, which approximates the country’s overall rural/urban population distribution. None of the households nor the individuals that comprise them are identifiable.

We use a sample of 6101 observations on 3427 different children age five or less over the three years of the LSMS survey. The children included in this sample reside in households for which complete data exist for all the variables used in our analysis. Sampling bias might exist if, for example, households with chronically ill members systematically do not answer questions about health. The UBOS staff employs sampling methods designed to mitigate this sort of systematic bias, so we have no reason to expect any. The datasets we compiled from the UNPS are available from the corresponding author upon reasonable request.

### Variable descriptions and statistical design

The outcome variables for stunting and wasting in our statistical analysis are defined according to the WHO’s Child Growth Standards, which were developed from the their Multicentre Growth Reference Study [39]. We compute a z-score (HAZ) for each child in our sample as the difference between the child’s height-for-age and the mean for all children of the same age and gender from the WHO reference group, normalized by the standard deviation of the reference group. The WHO standards use length for children less than 24 months old and height for those 24 months to 5 years of age. The child is defined as stunted if HAZ < −2. That is, a child is stunted if he or she is more than two standard deviations below the average height-for-age for the WHO’s global reference group. These are the shortest 2.3% for their age. Wasting is similarly defined as WHZ < −2 for a child’s weight-for-height/length.

Rather than using HAZ and WHZ as the measures of nutritional status in our regression analysis, we focus specifically on stunting and wasting as defined above. We thus use two separate binary logistic regressions to estimate the effect of a larger household SBS and other covariates on the probability that a child is stunted or wasted. So the outcome variable for the stunting regression is set equal to 1 if HAZ < −2 and 0 otherwise. Similarly, the outcome for the wasting equation equals 1 if WHZ < −2 and 0 otherwise. We employ the same method used in the study of Ethiopian children [33]. Imposing the logistic functional form requires transforming the outcome variable into the natural log of the odds that a child’s growth is stunted or he or she is wasting in order to estimate the following logit regression

1 $$ \ln \left(\frac{{\mathrm{p}}_{\mathrm{it}}}{1-{\mathrm{p}}_{\mathrm{it}}}\right)=\mathrm{f}\left(\mathrm{SBS},\mathrm{gender},\mathrm{household}\ \mathrm{composition},\mathrm{SES},\mathrm{food}\ \mathrm{insecurity},\mathrm{urban},\mathrm{region},\mathrm{time}\right)\kern1em $$

Where p_it_ and $\ln \left(\frac{{\mathrm{p}}_{\mathrm{it}}}{1-{\mathrm{p}}_{\mathrm{it}}}\right)$ are, respectively, the probability and the natural log of the odds that stunting or wasting is occurring for the i^th^ child in year t. This regression emphasizes that SBS, constructed from food spending data for the entire household, explains the stunting and wasting of individual children.

The regressions include a number of control covariates motivated by past work to better isolate the effect of SBS on the likelihood of stunting/wasting. Table 2 also provides definitions, summary statistics, and expected signs for all these. We include an indicator of the gender of each child in the sample [40]. The ‘household composition’ category includes indicators of whether a child’s biological parents are present (*father present* and *mother present*) because a very similar method finds that children in Western Kenya who do not live with their biological parents are at increased risk of being stunted [27]. We include the percentage of household members who are female (*percent female*) to distinguish between a child’s biological mother and other women in the household. We do this because it is typical for extended family members to share a dwelling, and because females are more likely to contribute to childcare. We also include an indicator to identify the gender of the *household head*. Because the household head can affect food purchase decisions, this should better isolate the importance of the biological mother and father alone independent of the household head. Following the studies cited above, ‘household composition’ also includes a measure of the educational achievement of the household head.^1^

The ‘SES,’ or socioeconomic status, variables include the number of *household occupants* and their *average age* to help better isolate the relationship between SBS and our measures of undernutrition. Because staple foods are inexpensive, a larger household might affect its SBS. Similarly, the average age of the household members can affect the household’s SBS if there are more children and/or older people who do not contribute to household wealth. In addition, younger people may simply require more calories per day, especially if they are engaged in physical labor.

‘SES’ also includes total household *spending*, which is commonly accepted as a proxy for household earned income, and is necessary because income data is available for only a small fraction of the households in our sample [41]. This is generally true in sub-Saharan Africa because labor income is sporadic. Spending has also been shown to be a reliable proxy for income there [42]. Spending helps capture household poverty, thus holding constant its effect on raising the SBS because staple foods are relatively inexpensive.

Related to spending, we also include a measure of *food insecurity* that indicates if a household reported not having enough food to feed the household in one or more months during the survey year. This will capture any effect on stunting/wasting from food being unavailable for both direct and indirect reasons. Direct effects would include drought, flooding, or pestilence, while indirect effects would include higher prices due to shortages for these and other reasons (e.g, higher fertilizer prices).

We include an *urban* indicator to capture any differences due to urban location. Indicators for the region in which each household is located are also included to approximately control for ethnic differences and for differences in the mix of staple foods a household consumes. Lastly, we include indicators for each of the three years the data encompass to capture time-varying unobserved heterogeneity.

We take advantage of the panel component of our data by estimating a random effects model to control for unobserved heterogeneity among the children in our sample. Random effects remove correlations between our covariates and these unobserved differences that would otherwise bias the estimated coefficients and odds ratios. This allows us to better isolate causal relationships between our covariates and stunting and wasting. We use random effects for two primary reasons. First, fixed effects estimation, the primary alternative means of mitigating bias with panel data, cannot estimate the effect of time-invariant covariates because fixed effects “demeaning” causes these covariates to vanish. Second, our panel is unbalanced. In fact, 42% of the children in the sample appear only once in the three-year period. This is mainly because the sample includes only children aged 5 or less. For example, 5-year olds in the first survey year drop out of subsequent years, and 1-year olds in the third year do not appear in the first two. Moreover, 7% of the children appear only in the middle year, which can occur if a child has moved to a different household or died. Constructing indicators for each child is implicit in fixed effects demeaning. Doing so for those who appear only once renders estimation impossible, and eliminating them would shrink our sample by 42%.

Because the coefficients from logistic regressions measure the change in $\ln \left(\frac{{\mathrm{p}}_{\mathrm{it}}}{1-{\mathrm{p}}_{\mathrm{it}}}\right)$, they lack readily intuitive explanations. We therefore convert them into adjusted odds ratios (ORs), which measure the change in the odds of stunting/wasting caused by a unit change in a covariate. They are computed as

2 $$ \frac{{\mathrm{e}}^{\upalpha +{\upbeta}_1{\mathrm{x}}_1+\dots +{\upbeta}_{\mathrm{j}}\left({\mathrm{x}}_{\mathrm{j}}+1\right)+\dots +{\upbeta}_{\mathrm{k}}{\mathrm{x}}_{\mathrm{k}}+\upvarepsilon}}{{\mathrm{e}}^{\upalpha +{\upbeta}_1{\mathrm{x}}_1+\dots +{\upbeta}_{\mathrm{j}}{\mathrm{x}}_{\mathrm{j}}+\dots +{\upbeta}_{\mathrm{k}}{\mathrm{x}}_{\mathrm{k}}+\upvarepsilon}}={\mathrm{e}}^{\upbeta_{\mathrm{j}}} $$

The denominator is the antilog of Eq. 1, which is the odds $\left(\frac{{\mathrm{p}}_{\mathrm{it}}}{{1-\mathrm{p}}_{\mathrm{it}}}\right)$ of a child having HAZ/WHZ < −2 given the estimated coefficients *β*_*k*_ and values of the covariates *x*_*j*_. The numerator, also the antilog of Eq. 1, is the odds of having HAZ/WHZ < −2 given a 1-unit increase of the *j* ^*th*^ covariate holding constant the others. The ratio of these two odds simplifies to ${\mathrm{e}}^{\upbeta_{\mathrm{j}}}$, which are simply the antilogs of the estimated coefficients.

## Results

**Fig. 2:**
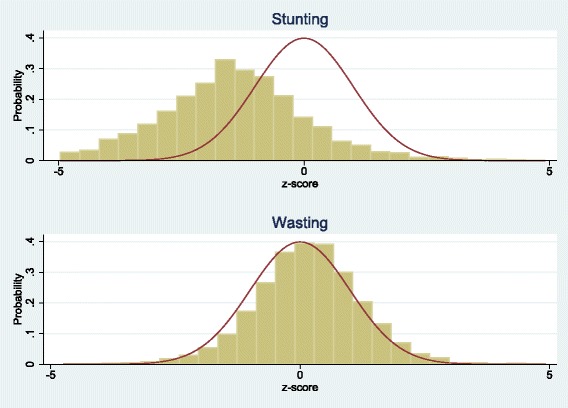
Probability Distributions of Z-Scores

Figure 2 shows histograms of the probability distribution of z-scores for our sample of Ugandan children. We superimpose the standard normal distribution of z-scores for the WHO reference group to allow a visual comparison of the differences. Clearly stunting is more prevalent because its histogram is shifted further to the left of the standard normal, which is centered on zero by definition. The mean values for stunting and wasting, reported in Table 2, show that stunting (22.2%) is indeed more prevalent than wasting (3.1%) in our sample.

We used Stata/SE Version 14.2 to estimate Eq. (1). Table 3 reports the estimated coefficients, 95% confidence intervals, and robust standard errors clustered at the person level for both the stunting and wasting regressions.^2^ We then divide the full sample into subsamples of children aged 12 months or less and those between 12 months and 5 years to test the robustness of our results. We also divide the full sample into male and female subsamples to test for gender differences. Table 4 reports the subsample analysis for both stunting (top panel) and wasting (bottom panel), with the results from Table 3 repeated for convenience. For simplicity, Table 4 only reports estimates that are statistically significant in one or more of the subsample regressions. As another specification check, we interact the age and gender subsamples with SBS in the full sample to test for an indirect effect of SBS on stunting and wasting. In all cases, we consider estimated coefficients significant at *p*-values ≤0.05.

**Table 3:** Logistic regression results, full sample

Number of obs (n): 6101				
Number of children: 3427				
	Stunting		Wasting	
	Coef. (std. error)	Odds Ratio (95% C.I.)	Coef. (std error)	Odds Ratio (95% C.I.)
SBS	0.007* (0.002)	1.007 (1.002, 1.011)	0.011* (0.005)	1.011 (1.001, 1.021)
Food insecurity	0.152 (0.098)	1.165 (0.960, 1.412)	0.332 (0.198)	1.393 (0.944, 2.056)
Spending	−0.005* (0.002)	0.995 (0.991, 0.998)	−0.001 (0.002)	0.999 (0.995, 1.004)
Household head	−0.075 (0.159)	0.928 (0.680, 1.266)	−0.151 (0.302)	0.860 (0.476, 1.553)
Average age	−0.008 (0.008)	0.992 (0.977, 1.007)	−0.010 (0.017)	0.990 (0.958, 1.023)
Household occupants	−0.029 (0.023)	0.971 (0.928, 1.016)	−0.048 (0.041)	0.953 (0.879, 1.034)
Percent female	0.000 (0.003)	1.000 (0.995, 1.005)	0.001 (0.005)	1.001 (0.991, 1.012)
Urban	−0.709* (0.169)	0.492 (0.354, 0.685)	−0.329 (0.306)	0.720 (0.395, 1.312)
Father present	0.205 (0.152)	1.227 (0.911, 1.653)	0.223 (0.308)	1.250 (0.684, 2.287)
Mother present	−0.325* (0.162)	0.723 (0.526, 0.992)	0.179 (0.340)	1.196 (0.615, 2.327)
Household head educated	0.009 (0.104)	1.009 (0.823, 1.238)	−0.402* (0.205)	0.669 (0.447, 1.000)
Male	0.404* (0.098)	1.498 (1.237, 1.815)	0.445* (0.184)	1.560 (1.087, 2.240)
Region Indicators	x		x	
Year Indicators	x		x	
Log pseudolikelihood	−3053.290		−807.927	
Wald X^2^ =	88.13		56.79	
Prob > X^2^ =	0.000		0.000	

*indicates *p* < 0.05. Heteroskedasticity-robust standard errors are clustered at the person-level. OR = e^coef^ = ratio of the odds of stunting/wasting after a unit increase in the corresponding covariate to the odds before the increase

**Table 4:** Logistic regression results, age and gender subsamples

Stunting										
	Full Sample		12mos or less		Greater than 12mos		Males only		Females only	
Number of obs (n)	6101		955		5146		3066		3035	
Number of children	3427		936		2993		1679		1751	
	Coef. (std. error)	Odds Ratio	Coef. (std. error)	Odds Ratio	Coef. (std. error)	Odds Ratio	Coef. (std. error)	Odds Ratio	Coef. (std. error)	Odds Ratio
SBS	0.007* (0.002)	1.007	−0.003 (0.006)	0.997	0.009* (0.003)	1.009	0.006* (0.003)	1.006	0.008* (0.004)	1.008
Spending	−0.005* (0.002)	0.995	−0.005 (0.004)	0.995	−0.005* (0.002)	0.995	−0.008* (0.002)	0.992	−0.003 (0.002)	0.997
Average age	−0.008 (0.008)	0.992	−0.015 (0.018)	0.985	−0.011 (0.009)	0.989	0.009 (0.009)	1.009	−0.034* (0.014)	0.967
Urban	−0.709* (0.169)	0.492	−0.481 (0.389)	0.618	−0.782* (0.196)	0.457	−0.849* (0.228)	0.428	−0.578* (0.248)	0.561
Mother present	−0.325* (0.162)	0.723	0.110 (0.773)	1.116	−0.205 (0.182)	0.815	−0.129 (0.218)	0.879	−0.558* (0.244)	0.572
Male	0.404* (0.098)	1.498	0.275 (0.249)	1.317	0.431* (0.114)	1.539	---	---	---	---
Log pseudolikelihood	−3053.290		−333.994		−2658.276		−1621.735		−1420.928	
Wald X^2^ =	88.13		8.46		86.76		48.18		42.14	
Prob > X^2^ =	0.000		0.956		0.000		0.0004		0.000	
Wasting										
	Full Sample		12mos or less		Greater than 12mos		Males only		Females only	
Number of obs (n)	6101		955		5146		3066		3035	
Number of children	3427		936		2993		1679		1751	
	Coef. (std. error)	Odds Ratio	Coef. (std. error)	Odds Ratio	Coef. (std. error)	Odds Ratio	Coef. (std. error)	Odds Ratio	Coef. (std. error)	Odds Ratio
SBS	0.011* (0.005)	1.011	0.015 (0.012)	1.015	0.008 (0.006)	1.008	0.006 (0.007)	1.006	0.017* (0.008)	1.017
Food insecurity	0.332 (0.198)	1.393	0.291 (0.451)	1.338	0.351 (0.225)	1.421	0.148 (0.261)	1.159	0.595* (0.298)	1.813
Household head educated	−0.402* (0.205)	0.669	−0.385 (0.440)	0.680	−0.357 (0.226)	0.700	−0.203 (0.268)	0.816	−0.660* (0.316)	0.517
Male	0.445* (0.184)	1.560	0.568 (0.448)	1.765	0.436* (0.206)	1.547	---	---	---	---
Log pseudolikelihood	−807.927		−200.675		−596.030		−463.902		−336.692	
Wald X^2^ =	56.79		6.12		45.08		34.9		30.55	
Prob > X^2^ =	0.000		0.992		0.000		0.004		0.015	

*indicates *p* ≤ 0.05. Each regression includes the same covariates as in Table 3 with the exception of the males only and females only regressions which did not include the gender indicator. For ease of exposition, only those covariates with significant coefficients in at least one regression are reported here. Complete results are available upon request. Heteroskedasticity-robust standard errors are clustered at the person-level. OR = e^coef^ = ratio of the odds of stunting/wasting after a unit increase in the corresponding covariate to the odds before the increase

Table 3 shows that SBS is associated with both increased stunting and wasting in the full sample. Moreover, Table 4 shows that SBS is associated with increased stunting in children between the ages of 12 months and 5 years, but not in children aged 12 months or less. Also, the subsamples divided by gender show that SBS is associated with increased stunting for both males and females, but with increased wasting only for females.

Table 4 also shows that a number of the other covariates are statistically significant. Greater household spending, our income proxy, is associated with lower stunting but not wasting. Male children more than 12 months old appear more prone to both stunting and wasting independent of SBS. Having the biological mother present is associated with less stunting, and a household head with some formal education is associated with less wasting both in the full and female only samples. Urban location is associated with a rather large decrease in stunting in all samples other than the one containing only children 12 months old or less. This result is particularly robust. Finally, we find that food insecurity affects only wasting in the female subsample.

## Discussion

Our results indicate that SBS is associated with both stunting and wasting. However, a comparison of Tables 3 and 4 shows that stunting is better explained by the set of covariates than is wasting. This is most likely because, as Fig. 2 shows, stunting is the larger problem in Uganda. This in turn is consistent with reports of a general decline in the prevalence of stunting in less developed countries except for those in East Africa [1, 43].

In the stunting regression, the odds ratio associated with the SBS coefficient is 1.007 (e^0.007^), which indicates that a 1 percentage point (equal to 1% here) increase in household SBS is associated with a 0.7% increase in the odds of a child being stunted. Alternatively, the odds of stunting are 7% higher for a household that spends 10% more of their food budget on staples, and the estimate is quite precise (standard error = 0.002, *p* = 0.005). Moreover, this finding for Ugandan children is consistent with the work that shows lower odds of stunting for Ethiopian children that have a more diverse diet [33]. It therefore also suggests that the proportion of staples is the complement to dietary diversity.

We also find a significant relationship between SBS and wasting, where a 10% increase in SBS is associated with an 11% increase in the odds of wasting (OR = 1.011, *p* = 0.034). This odds ratio is almost double that in the stunting regression. By itself, this appears odd given that Fig. 2 shows stunting to be the much larger problem. However, one should compare these odds ratios relative to the means for stunting and wasting in Table 2. For example, evaluated at the mean for stunting (22.2%), the 7% increase in the odds of stunting from a 10% increase in SBS would raise the mean from 22.2% to 23.8%. Evaluated at the much smaller mean for wasting (3.1%), the 11% increase in odds from a 10% increase in SBS raises the mean from 3.1% to only 3.4%. Thus, the larger odds ratio for wasting predicts a smaller increase in the average number of wasted children because this average is so much smaller.

Focusing on the subsamples, the SBS coefficient is not statistically significant in the stunting regression for children 12 months old or less (*p* = 0.587), but is again positive and statistically significant (*p* = 0.001) for those more than 1 year old, with a 10% greater SBS associated with a 9% increase in stunting (OR = 1.009). This is reasonable because many, and perhaps most, children are not yet eating solid food during their first year. Therefore, the statistically significant odds ratios for SBS suggest we are indeed seeing that staple foods affect stunting rather than some spurious correlation.^3^ Taken together, the SBS odds ratios in these two subsamples provide some evidence that stunting is not solely a function of maternal malnutrition. Nevertheless, the insignificant SBS indirectly suggests that maternal malnutrition is the cause of the stunting (11.8%) that does exist in this subsample if our list of covariates reasonably covers the other causes identified in past work. As another robustness check, we interact age (1 year or less or greater than 1 year) with SBS to test for an indirect effect of SBS on stunting and wasting. The combined odds ratio is 1.010 (*p* < 0.001) in both cases, which is virtually identical to the odds ratio in the 1-to-5 year old stunting subsample (1.009) and in the wasting subsample (1.008), although the latter is not significant.

Subsamples for males and females show that stunting is not gender-specific. The odds of male stunting increase 6% (OR = 1.006, *p* = 0.05) and the odds of female stunting increase 8% (OR = 1.008, *p* = 0.027) with a 10% increase in SBS. The same 10% increase in SBS also shows a 17% increase in the odds of wasting (*p* = 0.024) for females. This represents an increase to 3% from the 2.6% wasting mean in this subsample. Perhaps somewhat troubling, we find that food insecurity raises the odds of wasting by 81%, in this same subsample (OR = 1.812, *p* = 0.046). Even though only 2.6% of the females are wasted, this odds ratio implies female wasting increases to 4.7% if the household cannot provide enough food at some point during the survey year. One interpretation may be that when households cannot provide enough food, including what they grow themselves, young girls are deprived. Finally, we interact gender with SBS but find no significance.

Returning to the stunting equation using the full sample, the estimated coefficient for spending is negative, which indicates that greater household spending is associated with lower odds of stunting. The odds ratio, which is less than 1 (e^−0.005^ = 0.995), indicates that a 100,000 Uganda shillings (UGX) increase in spending is associated with a (1–0.995)×100, or 0.5%, lower odds of stunting (*p* = 0.005). For perspective, UGX 100,000 translates into about €31.88 ($42.81) at the average exchange rates over the sample time period.

We expect the negative relationship between spending and stunting because spending is a proxy for income and, other things the same, higher income should be associated with lower odds of stunting. This is consistent with the work that shows how household wealth affects stunting [15–17]. The FANTA 2 report states that low levels of zinc, common in these staple foods, are associated with stunting. It also reports that households spend on average only 54% of their budgets on food. This result, together with SBS, suggests that stunting is not solely, and maybe not even largely, a function of poverty.

We noted earlier that others have found greater stunting among male children [21]. We find that, independent of SBS, males have greater odds of both stunting and wasting in our full sample as well as our subsample of children more than 1 year old, yet we have no evidence from past work to suggest why. Moreover, the rather large increase in odds suggests that male is capturing other factors we do not measure. In addition, having the biological mother present appears to be important. Our finding that this lessens the odds of stunting is consistent with earlier research with the same finding [27].

The odds ratio for an urban location (0.492) indicates that the odds of stunting are 50.8% lower for children in urban households (*p* < 0.001). This is perhaps not surprising given that access to all sorts of information is much more readily available in cities, including access to neonatal and postnatal consultation with health care providers who no doubt also provide nutrition advice. The magnitude of this effect is large, however, which suggests the coefficient is capturing many attributes of urban areas that mitigate the likelihood of undernutrition. This indicator is particularly robust across the stunting regressions, which suggests another possible avenue for future research.

In passing, for completeness, we note that a third anthropometric measure of undernutrition is a child’s weight-for-age, with underweight defined as WAZ < −2. Weight-for-age has been described as a composite of height-for-age and weight-for-height, which makes its interpretation difficult [44]. As such, it is most commonly used to monitor children’s growth and to assess changes in the magnitude of undernutrition over time. Nevertheless, we estimate a weight-for-age logit as another test of the robustness of our other estimates. SBS significantly explains being underweight (OR = 1.0136, *p* < 0.001). These results are available from the authors upon reasonable request.

While we find strong evidence that staple food consumption is associated with stunting and wasting in young children, there are limitations to the analysis. First, because we focus solely on diets with larger proportions of staples, we ignore the complementary foods that complete the typical diet. Because a greater proportion of less nutritious staples strongly associates with the odds of stunting and wasting, households most likely consume a smaller proportion of more nutritious complementary foods such as those rich in vitamin A and protein. Yet the UNPS data also reports that Ugandans buy less-nutritious items that are not part of SBS such as sugar, sesame, coffee, tea, soda, beer and other alcohol, and restaurant food, which could be more or less nutritious depending on how it is prepared. The point is we cannot say that all the foods that complement the staples are more nutritious.

Second, we use SBS, a household-level measure of staple food spending, to explain the stunting and wasting of individual children. This implicitly assumes that nutrition content is equally distributed among all household members, which would be incorrect if, for example, children receive more nutrient-dense food than others do as part of an intra-household food allocation decision. While possible, a theoretical economic model of optimal household resource allocation in poor countries suggests the opposite is also possible. Although focused on a different health issue, this model predicts that households maximize well-being when they allocate all resources that promote health to the most productive household members, which are typically those who engage in physical labor [45]. Data from India support the hypothesis. In light of this, any intra-household allocation may give young children less nutritious food than those who work.

In addition, our sample includes children from across Uganda, so we define SBS broadly because we know the mix of staples consumed varies by region. While region indicators in the regression broadly capture cultural and dietary differences, we do not determine if the relationship between SBS and undernourishment varies by region, or if different staples have different impacts. Future work should be able to shed more light on these details. Related to this, we expect that child undernourishment is related to staple consumption in other countries and world regions. Because staples and their nutritional content certainly vary, we cannot assert that our findings for Uganda apply elsewhere.

## Conclusions

The Uganda Nutrition Action Plan’s definition of food insecurity includes the frequent consumption of nutritionally deficient staples. We find evidence that lends support to this tenet of the plan. Overall, we find that a greater proportion of less nutritious staple foods, as measured by our staple budget share, is strongly associated with higher probabilities of stunting and wasting in children between one and five years old. Moreover, we find no evidence that staple foods affect children less than one year old because, generally, they are not yet eating solid food. This lends confidence to the validity of the relationships we do find, and we hope that Ugandan policymakers find them useful as they develop future nutrition policy.

Our work also suggests some avenues for future research. Because our findings are specific to Uganda, we hope others will analyze the same relationships in other countries and world regions. In addition, as we mention, the size of the estimated odds ratios for a few of the control covariates are very large, which suggests they are capturing unmeasured attributes that bias our estimates. In particular, the large impact of our measure of food insecurity on the wasting of young girls stands out. More work should help extricate any confounding influences.

Beyond the effect of staple foods, we find that urban location can greatly lessen the likelihood of stunting. We also find that males are more prone to both stunting and wasting independent of staple foods. We hope this motivates research to discover why.

## Abbreviations

EA: Enumeration area
FANTA: Food and nutrition technical assistance
HAZ: Height-for-age z-score
LSMS: Living standards measurement study
MDG: Millennium development goals
OR: Adjusted odds ratio
SBS: Staple budget share
SCS: Staple calorie share
SES: Socioeconomic status
UBOS: Uganda Bureau of Statistics
UGX: Uganda shillings
UNAP: Uganda Nutrition Action Plan
UNPS: Uganda National Panel Survey
WAZ: Weight-for-age z-score
WHO: World Health Organization
WHZ: Weight-for-height/length z-score

## Footnotes

1 We use the education of the household head because only 5% of the biological mothers in the sample reported having attended or completed primary school or higher, with most of the others reporting they do not know if they completed any formal education. Estimates using mother’s education are similar to those we report for the household head, but we are not confident in their reliability. Those results are available upon request.

2 Robust standard errors correct for unequal regression error variance (heteroskedasticity). They are different (usually larger) than uncorrected standard errors when the error variance is correlated with the values of the covariates, which is commonly found in cross-sectional and longitudinal data such as ours. Similarly, clustering at person level adjusts the standard errors to account for correlations in the error terms for the same child across the 3-year panel. Both corrections make erroneous conclusions about statistical significance less likely.

3 We recognize that many children begin eating solid food between 6 and 12 months, but the data do not allow us to determine when each child starts eating solid food.
